# ExpressionDB: An open source platform for distributing genome-scale datasets

**DOI:** 10.1371/journal.pone.0187457

**Published:** 2017-11-02

**Authors:** Laura D. Hughes, Scott A. Lewis, Michael E. Hughes

**Affiliations:** Division of Pulmonary and Critical Care Medicine, Washington University School of Medicine, St. Louis, Missouri, United States of America; University of Connecticut, UNITED STATES

## Abstract

RNA-sequencing (RNA-seq) and microarrays are methods for measuring gene expression across the entire transcriptome. Recent advances have made these techniques practical and affordable for essentially any laboratory with experience in molecular biology. A variety of computational methods have been developed to decrease the amount of bioinformatics expertise necessary to analyze these data. Nevertheless, many barriers persist which discourage new labs from using functional genomics approaches. Since high-quality gene expression studies have enduring value as resources to the entire research community, it is of particular importance that small labs have the capacity to share their analyzed datasets with the research community. Here we introduce ExpressionDB, an open source platform for visualizing RNA-seq and microarray data accommodating virtually any number of different samples. ExpressionDB is based on Shiny, a customizable web application which allows data sharing locally and online with customizable code written in R. ExpressionDB allows intuitive searches based on gene symbols, descriptions, or gene ontology terms, and it includes tools for dynamically filtering results based on expression level, fold change, and false-discovery rates. Built-in visualization tools include heatmaps, volcano plots, and principal component analysis, ensuring streamlined and consistent visualization to all users. All of the scripts for building an ExpressionDB with user-supplied data are freely available on GitHub, and the Creative Commons license allows fully open customization by end-users. We estimate that a demo database can be created in under one hour with minimal programming experience, and that a new database with user-supplied expression data can be completed and online in less than one day.

## Introduction

The human genome project was largely completed using conventional Sanger sequencing [1]. This initiative and other genome sequencing projects in turn motivated the development of technologies underlying next-generation sequencing [2]. Originally used to sequence short strands of DNA in a massively parallel fashion [2,3], a notable feature of next-generation sequencing is its ability to measure gene expression by sequencing RNA samples that have been reverse transcribed into cDNA [4]. Using this approach, short sequenced reads are aligned to the donor organism’s genome and/or transcriptome, and expression levels are calculated based on the normalized number of reads aligning to a given transcript or feature [5]. Prior to the development of RNA sequencing (RNA-seq), the primary method for genome-wide expression analysis was microarray technology, which is limited by lower data resolution and reliance on pre-designed complement probes [4,6]. In contrast, high-throughput RNA-seq offers direct mRNA quantitation over a broad dynamic range. Data from our lab shows expression levels spanning six orders of magnitude [7], and many others see comparable dynamic ranges [5,6,8,9]. As such, RNA-seq eliminates dependence on potentially confounding probe hybridization, while capturing the expression of low-abundance transcripts at the single-base level [9] with technical reproducibility at least as high as conventional microarray approaches [10].

Applications of RNA-seq have had an enormous impact on modern biology [11]. RNA-seq has been used to systematically characterize transcriptional diversity in human tissues [12], model organisms [13], and cancer cells [14]. Given its high resolution and quantitative nature, RNA-seq has allowed researchers to gain insight into diverse biological phenomena including host/pathogen relationships [15], stem cell pluripotency [16], cortical neuron connectivity [17], and circadian rhythms [18]. However, the minimum skill sets required to perform an RNA-seq experiment are far from universal in the biological community including fluency with biostatistics, entry-level programming skills, and comfort working with datasets that are orders of magnitude larger than those typically encountered in the lab. Even conventional microarray experiments require computational sophistication beyond that found in many labs. For these reasons, a variety of tools have been developed that do not require pre-existing knowledge of programming or expertise in large data analysis. A notable example is RobiNA [19], a practical resource that allows automated quality control and differential expression analysis. Visualization of expression data—microarray, RNA-seq, and proteomics—has been likewise been enhanced by the development of online visualization tools such as the European Bioinformatics Institute’s Expression Atlas[20]. Taking this approach one step further, recently published web-based applications have made high-throughput RNA-seq visual-analysis tools accessible to new users [21–24].

Nevertheless, there is a need to democratize these web-based programs by bringing advanced data visualization and distribution features to users with minimal experience in programming and little or no budget for professional database engineers. The ultimate goal is to disseminate these data resources among the biology community, particularly for American scientists under the jurisdiction of United States Executive Order M13-13 that requires any data produced with federally funded research money must be released to the public. Therefore, the design of a flexible application program interface should (1) consider a multitude of applications in various research contexts, (2) be supported by thorough documentation, and (3) provide features that accommodate users of diverse backgrounds and skillsets. Moreover, these platforms will encourage open and reproducible research by providing a system to share results and analyses among researchers.

In this manuscript, we describe ExpressionDB, a fully customizable application for sharing large quantities of expression data. Using Shiny, a package developed by RStudio, and numerous data visualization packages, we created an interactive website for users to explore, filter, and download expression data. We developed ExpressionDB in R [25], a statistical language commonly used in bioinformatics. This platform can be deployed online or run locally through Shiny running in R. In either case, the underlying code used to generate the application can be shared through a project-hosting site like GitHub, which satisfies one of the many aspects of reproducible analysis[26]. The goal of ExpressionDB is to reduce one specific entry barrier that prevents many labs from performing large-scale expression profiling, thereby democratizing large-scale functional genomics. At the same time, this software also provides a platform for advanced users to customize analysis and distribution of data.

## Results and discussion

In designing a reusable interface for visualizing and sharing expression data, we sought to create a tool that (1) allows multiple comparisons of interest to biologists, (2) is reproducible and open, and (3) is sustainable and extendible. First, we will discuss the interface design and functionality of ExpressionDB. Next, we will discuss the underlying architecture in Shiny and R and potential customization. Last, we will outline the steps for a user to adapt ExpressionDB to a new dataset.

### Interface design

To illustrate the functionality of a webpage built with ExpressionDB, we have created a database using our unpublished RNA-seq data from various mouse muscle tissues (http://muscledb.org). Our design philosophy was to make a distribution platform based around the most common questions biologists ask when studying RNA profiling data, including: (1) What are the expression patterns of my genes of interest? (2) What are the expression patterns of genes in the pathway or biological function I study? We therefore prioritized two primary comparisons users would want to make: differences in expression levels between samples for one gene or many genes, and pairwise differences in gene transcripts between two samples.

**Fig 1:**
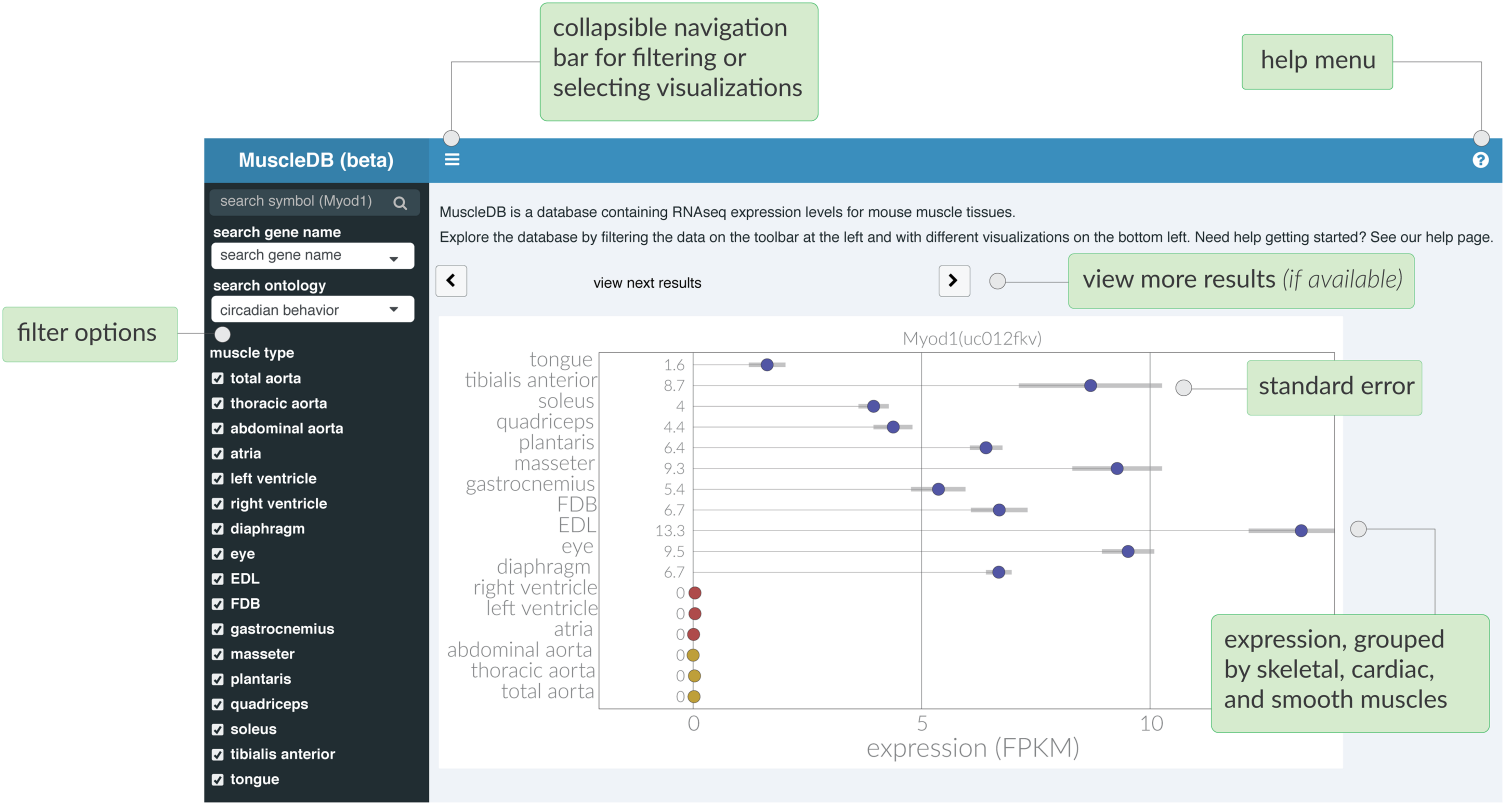
The ExpressionDB user interface is designed to showcase RNA-seq data with straightforward visuals. Dot plots represent expression levels of different transcripts in different samples, with error bars representing +/- S.E.M. Results can be filtered by gene symbol, gene name/description, or Gene Ontology (GO) terms.

The core purposes of ExpressionDB are to allow users to filter data to hone in on observations of interest; to interact with data, explore details upon demand, and analyze patterns; and to download data for further analysis. Looking at the front page (Fig 1), the user sees an overview of samples in the sidebar and their expression on the right in the form of dot plots. At the top left are two menus that allow users to search for gene symbols, gene names/description, and to filter the data based on gene ontology (GO) terms. In MuscleDB, for instance, there are over 40,000 transcripts in the database, so filtering based on gene name and/or functionality is essential to making sense of the large amount of data.

**Fig 2:**
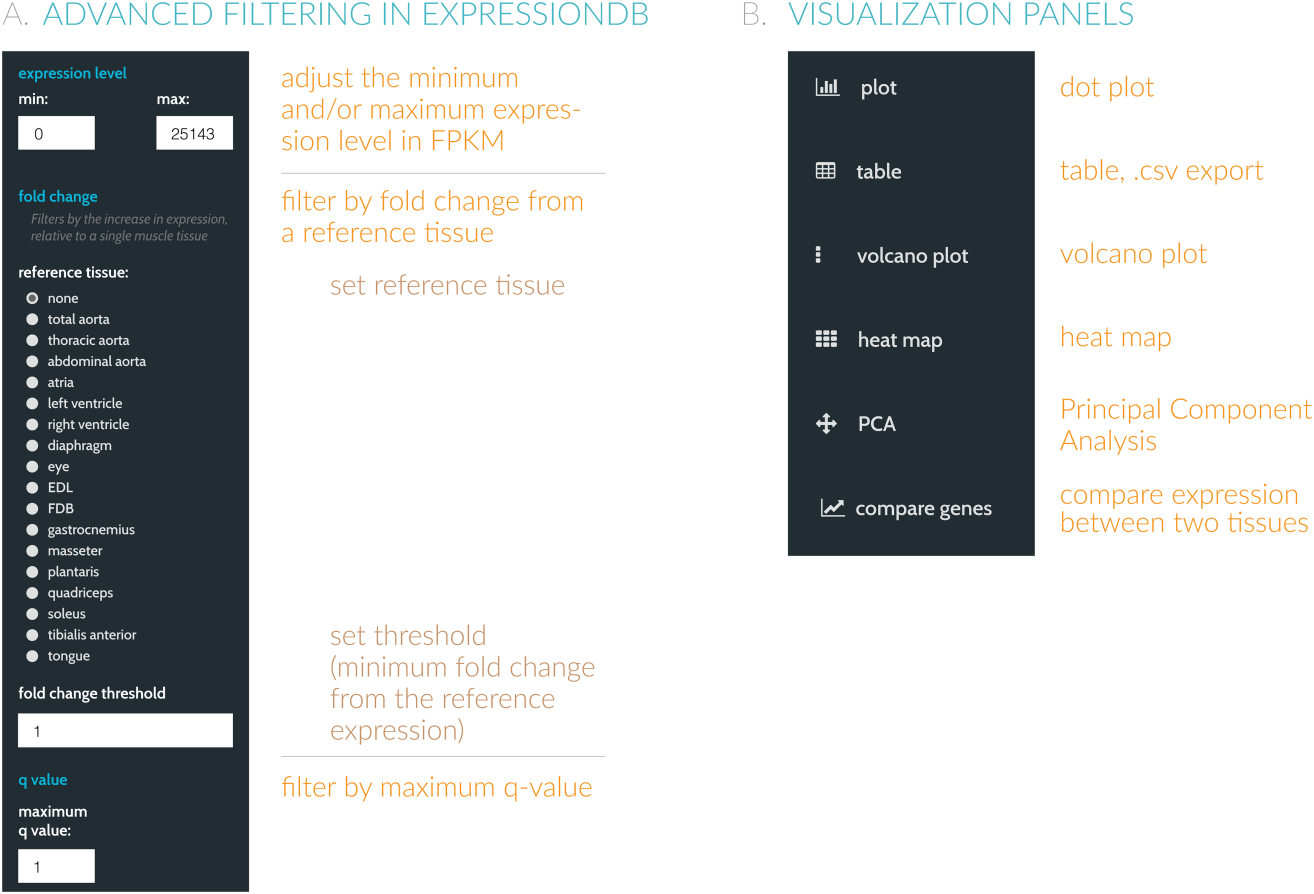
ExpressionDB supports a number of advanced options to filter data. (**A**) By ticking the “Advanced Filtering” option, the user may choose to examine ranges of expression levels, as well as choose the reference sample for calculating fold change between any two samples. ExpressionDB allows filtering based on q-values, allowing the user to browse through statistically significant features. (**B**) Additional visualization methods, including downloadable tables, heatmaps, and volcano plots can also be accessed here.

In addition, ExpressionDB includes advanced options (Fig 2) that allow users to dynamically filter their data using a variety of parameters. For example, Fig 2A shows a dialogue box that allows users to restrict which transcripts are displayed using expression level filters. Users can also filter by fold change relative to a particular sample to isolate transcripts that are up- or down-regulated compared to a reference. Users can filter their data by statistical significance (e.g. a maximum q-value threshold from an ANOVA test). Either before or after filtering, users can choose to inspect the data through a series of interactive visualizations including the default dot plot view, volcano plots, comparisons of similar genes, heatmaps, and principal component analysis (Fig 2B).

The default dot plot visualization shows the user a series of plots that allows the user to compare the variation in expression between samples. While the individual plots are all scaled to the same dimensions to enable comparison between plots, the focus is on the expression within a particular transcript or gene.

**Fig 3:**
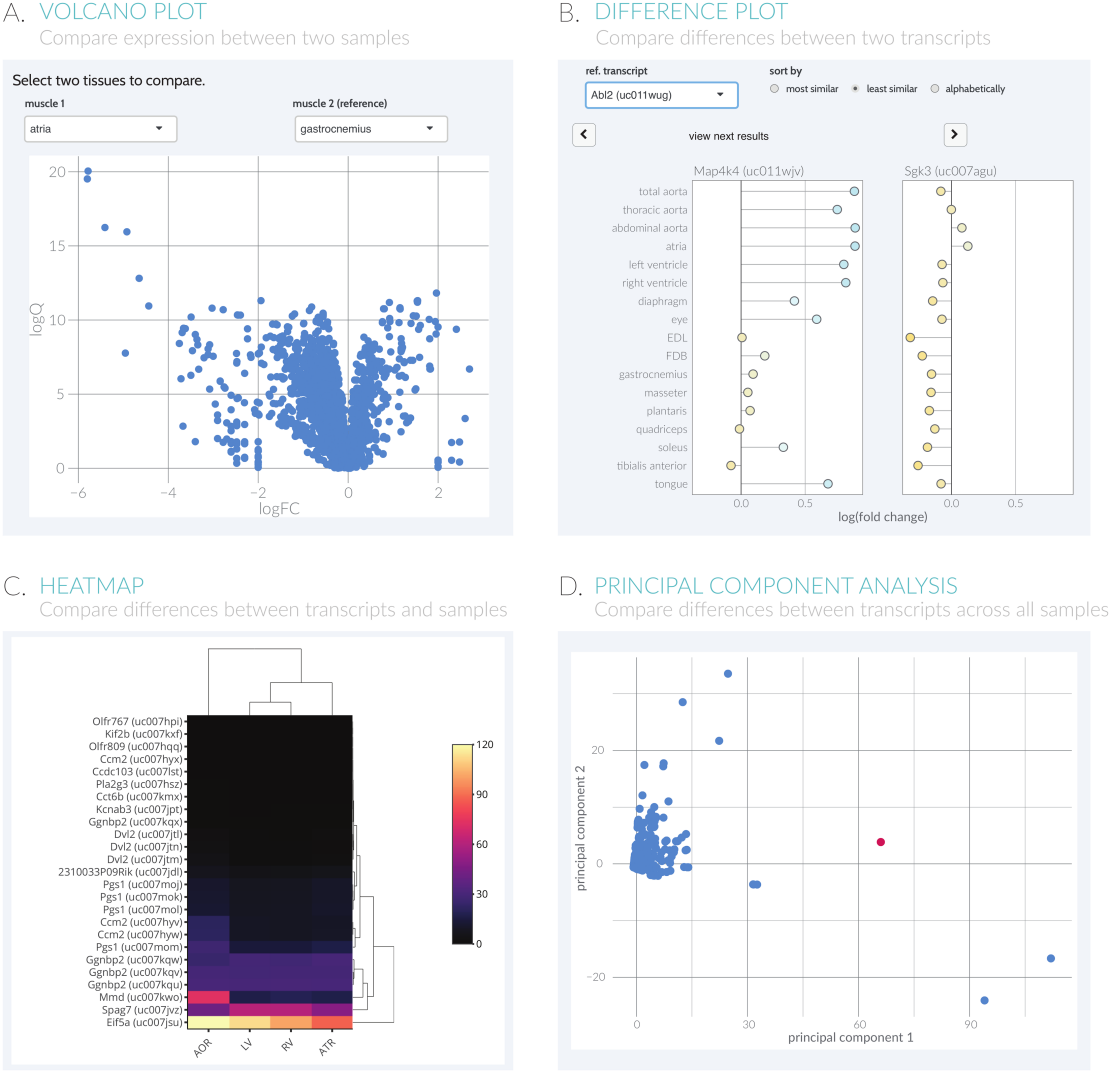
ExpressionDB supplies four built-in visualization methods. Example (**A**) Volcano Plots, (**B**) Gene Comparisons, (**C**) Heatmaps, and (**D**) Principal Component Analysis are shown here.

To allow for a more global view of all the transcripts within two samples, the volcano plot function (Fig 3A) allows users to look for outlier transcripts based on statistical significance and fold change when comparing two biological samples. This visualization allows users to ask questions such as “Which transcripts have significantly higher or lower expression compared to a reference sample?”Interactive plots allow users to identify differentially-expressed genes, zoom in on areas of interest, and export the raw data as necessary.

Instead of comparing expression between two samples to identify outlier genes, another user-identified need was the ability to compare differences between two transcripts. For instance, the user may want to know, “Are any kinase genes expressed similarly to the gene I study?” Within the Difference Plot view (Fig 3B), the fold change between all the transcripts and a reference are calculated and plotted in a dot plot. To facilitate easy comparison, the dots are colored by their difference in the log-fold change in expression, with blue dots showing the most up-regulated genes relative to the reference, yellow dots showing little difference, and red dots showing the most down-regulated genes. Users can then sort the plots either by transcripts that show the most or least similarity to the reference.

To compare a large number of transcripts and samples simultaneously, a heatmap option is provided (Fig 3C). Within the heatmap, transcripts are shown as rows, samples as columns, and the expression as a color ranging from black (low expression) to yellow (high expression). The heatmap function includes options to (1) normalize across transcripts (rows) or biological samples (columns), (2) hierarchically cluster by similarity across rows and columns, (3) log-transform the expression levels, and (4) zoom in on any given region of interest.

While the heatmap provides an overview of differences both in transcripts and samples, only the first fifty transcripts are shown to facilitate interpretability of the plot. If the user is interested in comparing more transcripts, a principal component analysis (PCA) plot is provided to visualize the similarities and differences between the different subgroups of a dataset (Fig 3D). Within the plot, a PCA is calculated on the fly for the selected observations, reducing all the samples into orthogonal principal components. The first two principal components are then plotted as a scatter plot, with each point representing an individual transcript. The PCA compresses the samples (e.g. tissues) from the heatmap into a new coordinate system that allows for easier identification of outliers across all the samples. In addition to the scatter plot, the PCA loadings for the first two principal components are shown, allowing the user to determine which samples are most responsible for the variation between transcripts.

Finally, all plots can be downloaded as Portable Network Graphics (.PNG) files for insertion into grant proposals, papers, or talks. To download the raw data and statistics, ExpressionDB includes a summary table that can be downloaded in Comma Separated Value (.CSV) files thereby facilitating custom offline analysis. Hyperlinks to Entrez Gene are conveniently embedded in the table to assist users exploring the function of differentially-regulated genes.

### Underlying architecture

ExpressionDB was built using Shiny, a web application framework in R (http://shiny.rstudio.com/). As such, users can quickly build web-based applications in native R without having to learn HTML5, Javascript, or CSS. This approach offers many advantages to visualize and to share data, especially when compared to conventional tools like Excel.

First, since it is built using R, ExpressionDB integrates with native R functionality, including the myriad statistical packages. This means that all analysis and development can be done in R: the raw data can be imported, statistics can be calculated via automated scripts, and an analysis platform can be created in ExpressionDB. Compared to Excel, the data manipulations are transparent, reproducible, and sustainable. For instance, if new data are collected, they merely need to be formatted in the same way for the expression averages, statistics, and visualizations to be recreated automatically. Not only does this save time, it means that the user can visualize thousands of data points in multiple ways quickly. Since ExpressionDB is open-source and freely available, the large R user base can extend it as appropriate.

Second, data visualizations within the ExpressionDB platform take advantage of the dynamic nature of websites. Working with large datasets, the challenge is often finding the signal within the noise: i.e. which observations are relevant and interesting to the user? Indeed, when creating a general resource to share RNA-seq data, users will have a multitude of purposes for analyzing the data. For instance, one user may be interested in expression of a small subset of transcripts, while another may be interested in the broader expression of a gene family, and yet another may focus on identifying how samples differ within the dataset rather than focusing on single transcripts. By placing these data within an interactive environment, the user can easily restrict their queries on-demand to only display the transcripts or samples relevant to their research.

Third, the flexibility of Shiny allows for applications to be deployed (1) locally on a personal computer, (2) hosted for free on the shinyapps.io website, or (3) hosted on a local or cloud-based server. In its simplest form, ExpressionDB can be launched on a personal computer through RStudio; all that is required is a local copy of the data, the ExpressionDB R scripts, RStudio / Shiny, and Chrome. This option does not require an internet connection, which makes it advantageous for doing live demos with unreliable connectivity, or in situations where data are sensitive and should not be made public. Additionally, running a local instance is ideal for testing and development. In our case, we choose to host our demo website on an Amazon Web Services cloud-based server, which can be scaled to meet the demands of the application.

### Customization

Our example database, MuscleDB, was built using RNA-seq data from an atlas of multiple muscle tissues. However, essentially any experimental design can be handled by ExpressionDB, including case/control experiments. Although we have tested this platform using RNA-seq alignments from RUM [27] and performed statistics using one-way ANOVAs of transcript-level FPKM expression, any user-supplied expression levels and corresponding statistical analyses are supported by this platform. We emphasize that these normalization methods and statistical analyses are not optimally powerful for all experimental designs. ANOVAs of FPKM-normalized data were chosen as the default because they are conceptually intuitive and maximize parallels with microarray experiments. We therefore encourage all users to explore best practices for the analysis of their experiments before uploading data to a public site. Related to this point, we encourage all users to seek out appropriate guidance either from the literature or from biostatisticians regarding the state-of-the-art for RNA-seq alignment, normalization, batch effect adjustments, etc., since the default parameters chosen here are intended to be illustrative, rather than bias users towards specific analytical approaches. Although it is not our intent to review systematically the best practices for genome-scale transcriptional profiling experiments, we briefly summarize the necessary steps in the remainder of this paragraph, and we point interested readers to our references that include more authoritative discussions of these topics. In order to ensure the quality of RNA samples, it is recommended that investigators verify that RNA samples are free of damage and contamination—a number of fluorescence detection and microfluidic electrophoresis-based technologies exist for this purpose[28]. The next step is cDNA library preparation. While the appropriate documentation for library preparation is provided with the respective kits sold by all major RNA-seq platforms, advanced users may choose to generate their own kits and corresponding protocols. Once the cDNA library has been sequenced, a number of alignment/statistical analysis software exist to facilitate the normalization, quantification, and analysis of raw RNA-seq [27,29,30].

To aid the user in creating their own ExpressionDB, we have made all source code publically available on GitHub (https://github.com/5c077/ExpressionDB) which includes an online tutorial with screenshots (https://github.com/5c077/ExpressionDB/wiki/User’s-Guide). Our in-house testing suggests that a demo webpage can be created with minimal programming experience in under one hour. A fully functional ExpressionDB with user-supplied data can be online in under a day.

Given ExpressionDB’s open source design, it has the versatility to permit users to expand its functionality with any third-party software written in R. For this reason, we have licensed ExpressionDB as Creative Commons Share-Alike (CC BY-SA), meaning that anyone can use and edit this code to whatever purpose as long as they reference the original authors. We believe this freedom of collaborative design will allow the ExpressionDB platform to grow to meet users’ needs that we cannot anticipate. Moreover, we provide multiple avenues through which ExpressionDB will continue to be refined and meet additional users’ needs. Beyond the Users’ Guide, we also provide a wiki page for documenting updates, answering frequently asked questions, and providing additional annotation files. As the ExpressionDB user-base grows, we anticipate this resource will become increasingly valuable to both new and advanced users.

### Adapting ExpressionDB to new data

Full details on data preparation and using ExpressionDB can be accessed at: https://github.com/5c077/ExpressionDB/wiki/User’s-Guide. The user needs to prepare expression data with multiple replicates; the ExpressionDB platform will then calculate average, standard error of the mean (SEM), and analysis of variation (ANOVA) statistics for each gene/transcript. A brief summary of the steps are presented here:

1. Collect and prepare the data. After collecting an RNA-seq or microarray dataset and measuring expression levels using appropriate normalization methods, the data need to be formatted in a [31] tabular format with each unique gene in a separate row. Each column needs to have specific name corresponding to the sample group along with its replicate number. After collection and manipulation to tabular format, the data will need to be saved in a.csv file; an example of this file format is shown in Table 1. Aside from the quantification and normalization of expression data, which in many cases is outsourced by small labs, the data preparation can be done using Microsoft Excel and requires no special programming expertise.
2. Download RStudio and install necessary packages. To test the application, the user needs to download R (https://cran.r-project.org/) and RStudio (https://www.rstudio.com/products/rstudio/download/), both of which are open-source software.
3. Download or create annotation files. For many common organisms—human, mouse, rat, fruit fly—we provide pre-made annotation files for download from GitHub (see Table 2 for an example). Advanced users may consult the Users’ Guide to create their own custom annotation files or submit a request that they be provided online.
4. Edit Global.R, the key code for ExpressionDB. Only four lines of code in Global.R must be edited to point ExpressionDB to the data and annotation files, and to ensure that all sample names are correct.
5. Run Global.R in RStudio. The first time Global.R is run, it will download required R packages to run ExpressionDB. It will also calculate average expression per gene, SEM per gene, and will generate q values for differential expression of all pairwise combinations of tissues. An experiment-wide ANOVA for all samples will also be calculated. These values will be saved as a look-up table for all subsequent instances of the user-generated ExpressionDB. Likewise, the annotation and data files will be merged together using official gene symbol as a unique index. Users wishing to apply custom statistics may consult the Users’ Guide for additional details.
6. Customize the application. If desired, add in additional analyses, visualizations, or other functionality as needed.
7. Deploy the application. Release the application to collaborators and/or the public, either by sharing the code and data, deploying to shinyapps.io, or to a local or cloud-based server.

**Table 1:** Representative sample of the data file required to input user-specific data into ExpressionDB. This example includes two tissues with three replicates apiece downloaded from GTEx. Complete.csv file here: https://github.com/5c077/ExpressionDB/tree/master/data.

gene	STOMACH1	STOMACH2	STOMACH3	SALIVARY_GLAND1	SALIVARY_GLAND2	SALIVARY_GLAND3
**A1BG**	**262**	**236**	**284**	**267**	**533**	**324**
**A1BG-AS1**	**84**	**91**	**87**	**75**	**142**	**133**
**A1CF**	**2**	**2**	**2**	**6**	**1**	**2**
**A2M**	**68603**	**126956**	**144344**	**245041**	**138640**	**81812**
**A2M-AS1**	**265**	**132**	**185**	**402**	**294**	**484**
**A2ML1**	**50**	**7**	**30**	**57**	**29**	**61**
**A2MP1**	**44**	**4**	**6**	**13**	**32**	**35**
**A3GALT2**	**5**	**5**	**7**	**1**	**1**	**8**
**A4GALT**	**3597**	**3898**	**4646**	**2722**	**4655**	**3125**
**A4GNT**	**13**	**2**	**25**	**23**	**2**	**24**
**AA06**	**0**	**0**	**0**	**0**	**0**	**0**
**AAAS**	**2284**	**2216**	**3197**	**3185**	**1568**	**3266**
**AACS**	**1657**	**1008**	**1785**	**861**	**2571**	**1164**
**AACSP1**	**0**	**0**	**2**	**0**	**0**	**0**
**AADAC**	**0**	**5**	**0**	**2**	**6**	**8**
**AADACL2**	**0**	**0**	**0**	**0**	**0**	**0**
**AADACL3**	**2**	**0**	**0**	**0**	**6**	**0**
**AADACL4**	**4**	**4**	**2**	**3**	**2**	**6**
**AADACP1**	**7**	**12**	**2**	**0**	**83**	**0**
**AADAT**	**246**	**421**	**639**	**574**	**479**	**543**
**AAED1**	**1074**	**740**	**1083**	**1033**	**1502**	**643**
**AAGAB**	**1109**	**1628**	**1740**	**1192**	**1425**	**1443**
**AAK1**	**1032**	**501**	**590**	**393**	**790**	**576**

**Table 2:** Representative sample of the annotation file required to input user-specific data into ExpressionDB. This example comprises human annotations downloaded from Entrez Gene. Complete.csv files in appropriate format for many common organisms studied can be downloaded here:https://github.com/5c077/ExpressionDB/tree/master/data.

geneLink	GO	Symbol	description
https://www.ncbi.nlm.nih.gov/gene/?term=219464	**G-protein coupled receptor activity**	**OR5T2**	**olfactory receptor family 5 subfamily T member 2**
https://www.ncbi.nlm.nih.gov/gene/?term=219464	**olfactory receptor activity**	**OR5T2**	**olfactory receptor family 5 subfamily T member 2**
https://www.ncbi.nlm.nih.gov/gene/?term=219464	**plasma membrane**	**OR5T2**	**olfactory receptor family 5 subfamily T member 2**
https://www.ncbi.nlm.nih.gov/gene/?term=219464	**G-protein coupled receptor signaling pathway**	**OR5T2**	**olfactory receptor family 5 subfamily T member 2**
https://www.ncbi.nlm.nih.gov/gene/?term=219464	**integral component of membrane**	**OR5T2**	**olfactory receptor family 5 subfamily T member 2**
https://www.ncbi.nlm.nih.gov/gene/?term=219464	**detection of chemical stimulus involved in sensory perception of smell**	**OR5T2**	**olfactory receptor family 5 subfamily T member 2**
https://www.ncbi.nlm.nih.gov/gene/?term=390154	**G-protein coupled receptor activity**	**OR5T3**	**olfactory receptor family 5 subfamily T member 3**
https://www.ncbi.nlm.nih.gov/gene/?term=390154	**olfactory receptor activity**	**OR5T3**	**olfactory receptor family 5 subfamily T member 3**
https://www.ncbi.nlm.nih.gov/gene/?term=390154	**plasma membrane**	**OR5T3**	**olfactory receptor family 5 subfamily T member 3**
https://www.ncbi.nlm.nih.gov/gene/?term=390154	**G-protein coupled receptor signaling pathway**	**OR5T3**	**olfactory receptor family 5 subfamily T member 3**
https://www.ncbi.nlm.nih.gov/gene/?term=390154	**integral component of membrane**	**OR5T3**	**olfactory receptor family 5 subfamily T member 3**
https://www.ncbi.nlm.nih.gov/gene/?term=390154	**detection of chemical stimulus involved in sensory perception of smell**	**OR5T3**	**olfactory receptor family 5 subfamily T member 3**

## Methods

ExpressionDB is based in RStudio and uses the ShinyApps package in R, a platform that has made web application development feasible for researchers with little to no *a priori* experience in programming. Versions of all software packages used are shown in Table 3.

**Table 3:** Versions of all software packages used in developing ExpressionDB. Package versions can also be found online: https://github.com/5c077/ExpressionDB/tree/master/data.

Software	Version
**data.table**	**1.10.4**
**dplyr**	**0.7.2**
**DT**	**0.2**
**dtplyr**	**0.0.2**
**ggplot2**	**2.2.1**
**heatmaply**	**0.10.1**
**R**	**3.4.0**
**RColorBrewer**	**1.1–2**
**Rstudio**	**1.0.153**
**shiny**	**1.0.3**
**shinydashboard**	**0.6.1**
**stringr**	**1.2.0**
**tidyr**	**0.6.3**

We have tested ExpressionDB in R using Macintosh (OS Sierra), PC (Windows 10), and Linux (Ubuntu 12.04 or later) operating systems. We have tested all functionality on exemplar MuscleDB data (17 samples; > 40,000 transcripts apiece) on a typical Mac laptop (3.5 GHz processor, 16 GB RAM) with no difficulty. Users posting their data to the web will need to determine the number of concurrent sessions they expect to support in order to gauge an appropriate hardware footprint.

The app may be hosted at no cost on a shinyapps.io site (https://www.shinyapps.io/) or on a personal server using ShinyServer (https://www.rstudio.com/products/shiny/download-server/). Additional information and a detailed users’ guide to preparing data for upload can be accessed at https://github.com/5c077/ExpressionDB/wiki/User’s-Guide. Questions or comments may be submitted to our discussion board at https://github.com/5c077/ExpressionDB/issues.
